# Culture Models for Studying Thyroid Biology and Disorders

**DOI:** 10.5402/2011/275782

**Published:** 2011-07-12

**Authors:** Shuji Toda, Shigehisa Aoki, Kazuyoshi Uchihashi, Aki Matsunobu, Mihoko Yamamoto, Akifumi Ootani, Fumio Yamasaki, Eisuke Koike, Hajime Sugihara

**Affiliations:** ^1^Department of Pathology & Microbiology, Faculty of Medicine, Saga University, Nabeshima 5-1-1, Saga 849-8501, Japan; ^2^Department of Internal Medicine, Faculty of Medicine, Saga University, Nabeshima 5-1-1, Saga 849-8501, Japan; ^3^University Hospital Pathology, Faculty of Medicine, Saga University, Nabeshima 5-1-1, Saga 849-8501, Japan; ^4^Department of Surgery, Koike Hospital, Saga 840-0862, Japan; ^5^School of Rehabilitation Science, International University of Health and Welfare, Fukuoka 831-8501, Japan

## Abstract

The thyroid is composed of thyroid follicles supported by extracellular matrix, capillary network, and stromal cell types such as fibroblasts. The follicles consist of thyrocytes and C cells. In this microenvironment, thyrocytes are highly integrated in their specific structural and functional polarization, but monolayer and floating cultures cannot allow thyrocytes to organize the follicles with such polarity. In contrast, three-dimensional (3-D) collagen gel culture enables thyrocytes to form 3-D follicles with normal polarity. However, these systems never reconstruct the follicles consisting of both thyrocytes and C cells. Thyroid tissue-organotypic culture retains 3-D follicles with both thyrocytes and C cells. To create more appropriate experimental models, we here characterize four culture systems above and then introduce the models for studying thyroid biology and disorders. Finally, we propose a new approach to the cell type-specific culture systems on the basis of *in vivo* microenvironments of various cell types.

## 1. Introduction

**Figure 1:**
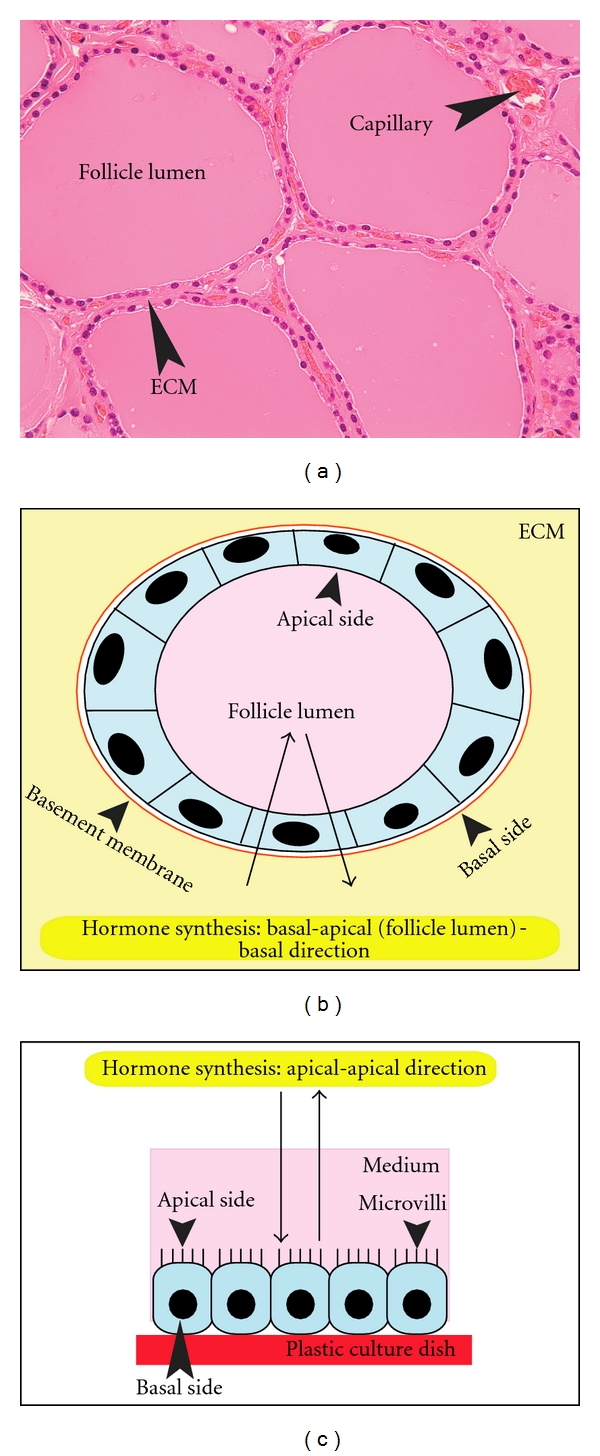
(a) Histology of the thyroid gland, comprised of colloid-filled thyroid follicles, which are supported by extracellular matrix (ECM) and a capillary network. (b) A schema of a thyroid follicle *in vivo* and in 3-D collagen gel culture. The follicle is embedded in ECM. The component thyrocytes of the follicle show a specific cellular polarity: the apical side with microvilli faces the follicle lumen, and the basal pole with basal lamina faces the ECM. Thyrocytes undergo thyroid hormone synthesis and release in a basal-apical (follicle lumen)-basal direction. (c) A schema of thyrocytes in a monolayer culture system. The cells have a cellular polarity: the apical pole with microvilli faces culture medium, and the basal side without basal lamina faces the plastic surface. Thyrocytes in such an environment carry out thyroid hormone synthesis and release only in an apical-apical direction.

Thyroid gland is composed of spheroid structures called thyroid follicles (Figure 1(a)), which consist of both many thyrocytes and a few C cells. Each follicle, which is an essential structural and functional unit of the thyroid, is supported by the stroma that contains interfollicular extracellular matrix (ECM), a capillary network and a few stromal cell types such as fibroblasts and inflammatory cells (Figure 1(a)). Thyrocytes have specific structural polarity: their apical pole with numerous microvilli faces the follicle lumen, and their basal side with basal lamina faces the stroma (Figure 1(b)). This is a specialized structure, compared to other endocrine organs, and results in thyroid hormone biosynthesis and release in a basal-apical (follicle lumen)-basal direction by thyrocytes (Figure 1(b)) [1].

To investigate both thyroid biology and diseases, monolayer and floating culture systems have been developed and widely used [2–5]. These methods have certainly facilitated the above-mentioned issues of the thyroid. However, the conventional methods, in which thyrocytes are unable to organize follicle structures, cannot satisfactorily provide thyrocytes with normal cellular integration [2–5]. In contrast, three-dimensional (3-D) collagen gel culture system allows thyrocytes to achieve follicle structures with their physiological polarity. This method is, thus, suitable for studying the normal and pathologic behavior of thyrocytes in a microenvironment which more closely simulates physiological conditions [4, 5]. These culture systems can not, however, reconstruct follicle structures that consist of both thyrocytes and C cells. To overcome this issue, we developed a new organotypic culture system of thyroid tissue fragments that contain the two cell types, using a concept of 3-D air-liquid interface (ALI) [6–8]. Our thyroid tissue-organotypic culture system retains 3-D follicle structures with both thyrocytes and C cells for a long term [6–8]. Given that highly integrated thyrocytes function to maintain body homeostasis through their intercommunication with neighboring thyrocytes, C cells, the other cell types, ECM molecules, and cytokines, the highly integrated thyrocyte-based experimental system seems critical for investigating both thyroid biology and disorders.

First, we review the characteristics of four culture systems above in order to create more appropriate experimental models. Second, we introduce some experimental culture models regarding the studies of the biology, regeneration, and diseases of the thyroid. Finally, we propose a new approach to the cell type-specific culture systems on the basis of *in vivo* microenvironments of various cell types.

## 2. Monolayer and Floating Culture Systems [2–5]

Thyrocyte monolayer culture initiated by Pulvertaft et al. in 1959 [2] has been used for studying the proliferation and differentiation of thyrocytes. However, monolayer culture cannot satisfactorily enable thyrocytes to achieve normal structural and functional polarities. In this culture system, thyrocytes organize a continuous epithelial pavement, adhering to the surface of the plastic dish (Figure 1(c)), and they show apical-basal polarity, with their apical side with microvilli facing the culture medium, and the basal (attached) side without basal lamina facing the plastic surface of the culture dish. In the epithelial sheet, some thyrocytes organize dome-like structures. The elevation of the cells from the plastic surface results in the formation of these structures, although the exact mechanism by which this occurs remains unclear. Thyrocytes covering these structures show microvilli on the side which contacts the culture medium, and they form foot processes on the luminal side. The plastic surface just under these structures is comprised of an acellular area.

In floating culture, thyrocytes organize themselves into inside-out follicles (vesicles), but not epithelial sheets, although they do form a continuous monolayer pavement on the plastic dish after long-term culture through their production of ECM molecules. The component cells of the vesicles have microvilli on the side that contacts the culture medium, and they have foot processes at the luminal side. In addition, the differentiating factor thyrotropin (TSH) transiently induces polarity inversion in the cells. In contrast, inside-out follicles undergo polarity inversion in a TSH-independent manner when vesicles are embedded and cultured within 3-D collagen gel. This suggests that a 3-D environment of ECM molecules is more important for the stability of thyrocyte integration than the addition of soluble TSH.

In these conventional systems, thyrocytes do not independently undergo thyroid folliculogenesis as is observed in 3-D collagen gel culture. After isolated thyrocytes are seeded in the conventional systems, the cells easily regain apical-basal polarity, as described above. In these conditions, thyrocytes do not require further polarization. In contrast, thyrocytes embedded in collagen gel are completely deprived of cellular polarity as described below. To regain normal cellular polarity, the cells must reconstruct 3-D follicle structures, in which the cells can undergo physiological polarization. Therefore, the essential reasons that there is no organization of thyroid folliculogenesis in conventional culture systems are considered to be: (1) that thyrocytes have apical-basal polarity as an epithelial phenotype even under an environment without follicle structures and (2) thyrocytes have no 3-D ECM environment. The monolayer and floating culture systems are certainly considered to be useful for simply investigating both the proliferation and differentiation of thyrocytes, but these systems seem inappropriate for studying these issues in a more physiological way.

## 3. 3-D Collagen Gel Culture System [4, 5]

**Figure 2:**
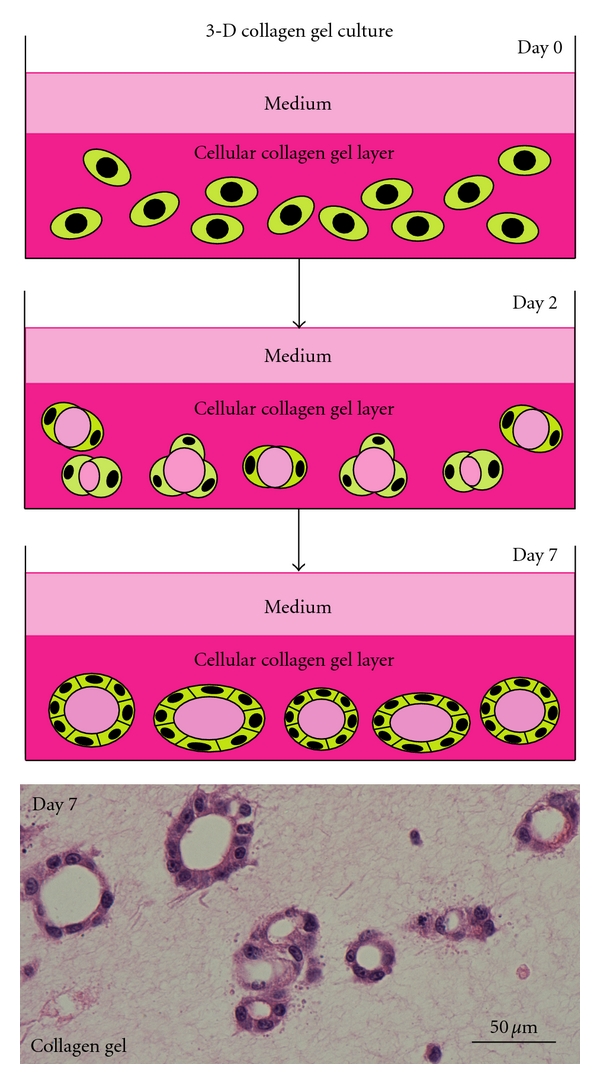
Scheme of 3-D collagen gel culture system and histology of thyroid follicles reconstructed in its system. Thyrocytes embedded in 3-D type I collagen gel (day 0) reconstruct small thyroid follicles (day 2). Thyroid follicles grow larger through proliferation of component thyrocytes (day 7). The lowest panel shows the histology of reconstructed thyroid follicles. Hematoxylin and eosin (H&E) stain.

**Figure 3:**
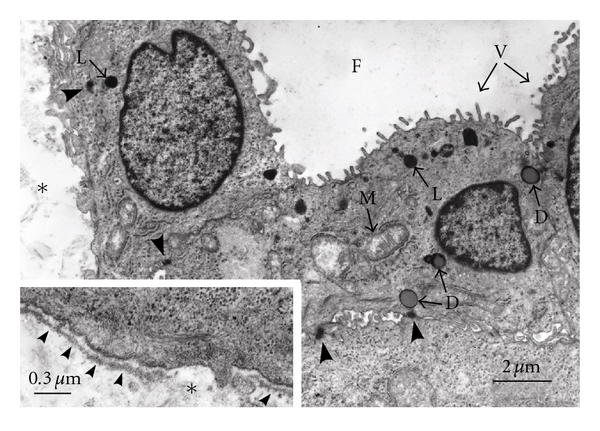
Fine structures of thyroid follicles reconstructed in 3-D collagen gel culture. The thyrocytes have numerous microvilli (V) at the apical surface of the follicle lumen (F) and form basal lamina (small arrowheads in inset) at the basal side which makes contact with collagen gel (∗). The cells have lysosomes (L), colloid droplets (D), and mitochondria (M). Junctional complexes (large arrowheads) are organized at the contact points of the cells.

Using the major component of ECM, type I collagen, Elsdale and Bard initiated collagen gel culture in 1972 [9]. Thyrocytes embedded in a collagen gel (Figure 2) reconstruct follicle structures, in which they show physiological cellular polarity (Figure 1(b)). They have numerous microvilli at the apical surface of the follicle lumen, and they form basal lamina at the contact side with collagen gel (Figure 3). This structural polarity allows thyrocytes to undergo thyroid hormone biosynthesis and release in a basal-apical (follicle lumen)-basal direction by the component thyrocytes of reorganized follicles (Figure 1(b)). The organization of this structural and functional cellular polarity is important for the homeostasis of normal thyrocytes. Thyrocytes cultured in a monolayer on a layer of collagen gel cannot form thyroid follicles. The 3-D collagen gel culture system enables thyrocytes to simulate *in vivo* cellular integration and behavior. This method could be successfully applied to the following experiments: (1) thyroid folliculogenesis; (2) functional actions of thyrocytes with stimulation of various factors such as TSH, iodide, methimazole, propylthiouracil, and cytokines; (3) interaction between thyrocytes and C cells, or mesenchymal cell types such as endothelial cells, fibroblasts, and inflammatory cells, involving experimental models for autoimmune thyroid diseases and cancer; (4) thyrocyte-ECM interaction; (5) cell transplantation. This method could also be applied to the investigation of other endocrine cell types, including pancreatic islet, adrenocortical, parathyroid, and pituitary cells, and adipocytes.

## 4. Thyroid Tissue-Organotypic Culture System [6–8]

**Figure 4:**
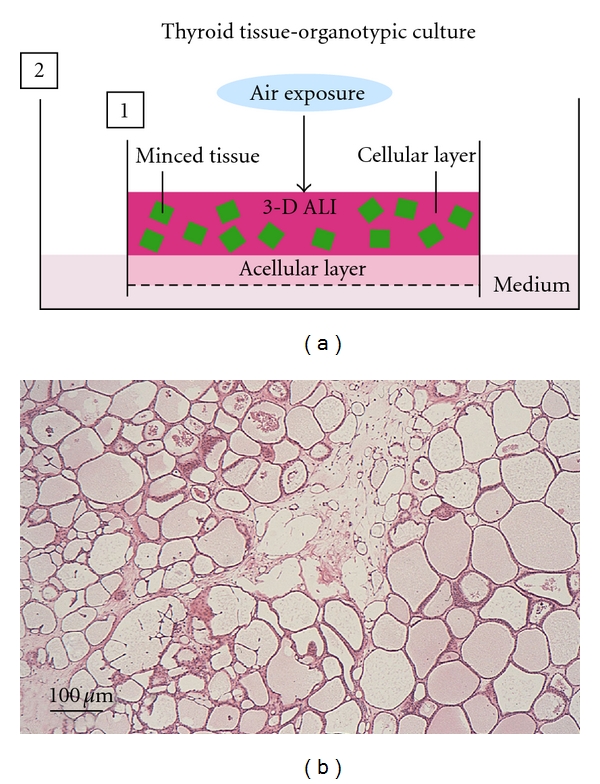
Scheme of thyroid tissue-organotypic culture system and histology of thyroid tissue maintained in its system. (a) Minced tissues embedded in type I collagen gel (cellular layer) are placed on the acellular gel (acellular layer) in inner dish (1). The inner dish (1) is put in the outer dish (2) with culture medium. In this way, the tissues in cellular layer are localized under air exposure-induced oxygenation (3-D ALI). The tissues are kept in moist and fed by culture medium that percolates by capillary action from the medium-containing outer dish, through the acellular layer and into the cellular layer. (b) Many viable thyroid follicles are maintained at 3 months in this culture system. H&E stain.

Thyroid follicles have two specialized cell types: principal thyrocytes and a few C cells (parafollicular cells). However, such viable follicles consisting of the two cell types cannot be organized and retained for a long term in any conventional monolayer, floating, 3-D collagen gel, and organ cultures. The follicles *in vivo* are embedded by interfollicular ECM, supported by a dense network of capillaries. This suggests that both 3-D ECM and sufficient oxygen supply are critical for the maintenance of follicular structure and function. By simulating this *in vivo* microenvironment of the follicles, we have established a new organotypic culture using a 3-D collagen gel culture of thyroid tissue fragments with improved oxygenation through air exposure-induced air-liquid interface (ALI) (Figure 4).

**Figure 5:**
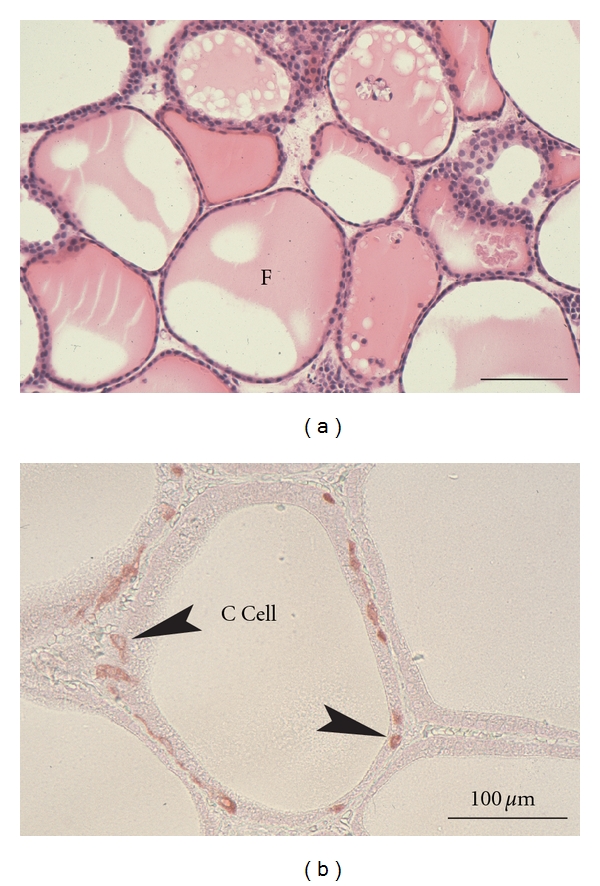
Histology of thyroid tissues (a) and immunohistochemistry for calcitonin (b) in thyroid tissue-organotypic culture. (a) At 40 days in culture, viable thyroid follicles enclosed by thyrocytes contain colloid substance in their lumens (F). H&E stain. (b) At 30 days in culture, thyroid follicles consisting of both thyrocytes and calcitonin-positive C cells (arrowheads) are clearly maintained.

In this system, viable 3-D follicles within thyroid tissue fragments are maintained for more than 6 months (Figures 4 and 5(a)). These follicles consist of both thyrocytes and C cells with their specific differentiation (Figure 5(b)). In the tissue periphery, thyrocytes actively undergo the growth and mother (primary) follicle-derived thyroid folliculogenesis. Likewise, isolated or clustered thyrocytes, which are located in the tissue periphery at the starting time of the culture, organize follicles. C cells show no proliferative ability and cannot grow even with the stimulation of various concentration of free calcium. This suggests that C cells may undergo terminal differentiation. A capillary network gradually disappears within the tissue fragments. Given that thyroid tissue-organotypic culture system retains the functional thyroid follicles with both thyrocytes and C cells for more than 6 months, this method is useful for analyzing the roles of thyroid gland in the biological behaviors of various functional cell types.

## 5. Experimental Culture Systems

As described above, thyrocytes are highly integrated within the follicles of thyroid gland. Thus, the highly integrated thyrocyte-based experimental system seems critical for investigating both thyroid biology and disorders in a more physiological way [1]. To facilitate the studies of thyroid biology and disorders, we here introduce such experimental models for analyzing thyroid tissue regeneration, thyrocyte-other cell type interaction, and thyroid tissue-other cell type interaction. 

### 5.1. A Model for Thyroid Tissue Regeneration

**Figure 6:**
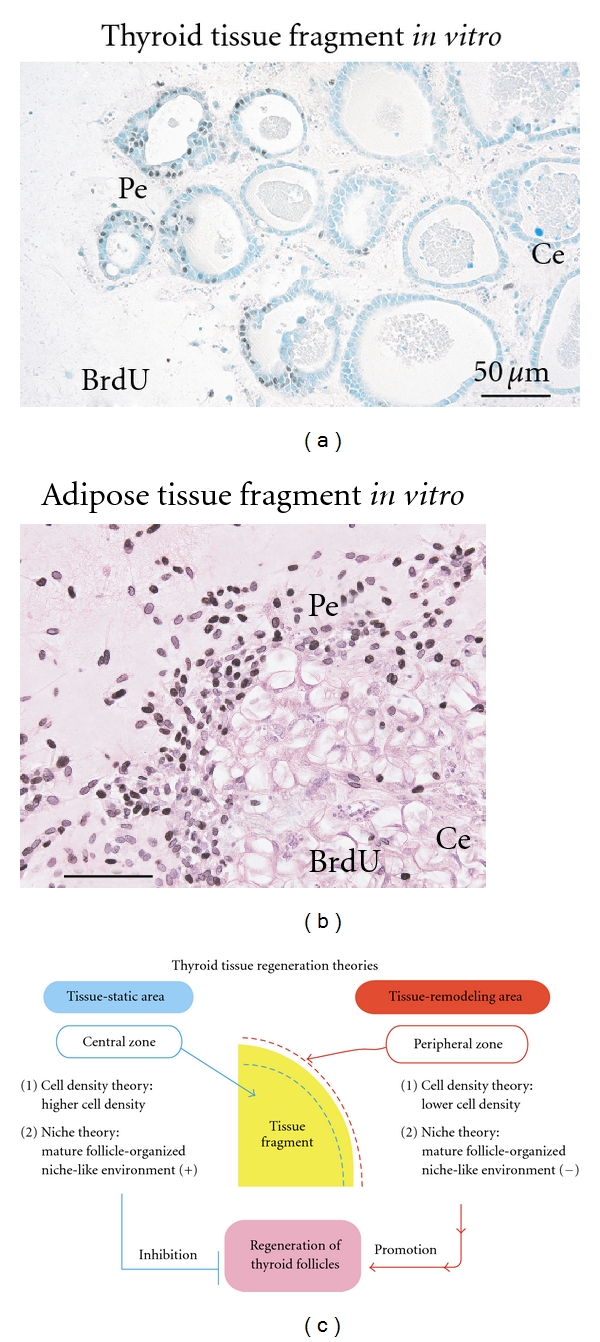
Immunohistochemistry with the growth marker BrdU in the organotypic cultures of thyroid (a) and adipose (b) tissue fragments, and scheme of thyroid tissue regeneration theories (c). (a) and (b) Intranuclear BrdU uptakes of thyrocytes and adipose cell types at peripheral zone (Pe) of thyroid (a) and adipose tissue fragments (b) are extensively greater than those at the central zone (Ce). Pe: peripheral zone. Ce: central zone. (c) Two theories regarding the mechanism of thyroid follicle regeneration that occurs specifically at peripheral zone of the tissue fragments. In regard to “cell density theory”, central zone of thyroid tissue fragments is characterized by higher cell density, whereas the peripheral zone is characterized by lower cell density. In general, increased cell density in a microenvironment inhibits the regeneration and growth of cells that are subjected to contact the inhibition of cell growth. Namely, central zone is tissue-static area with cell growth inactivation, while peripheral zone is tissue-remodeling area with cell growth activation. Thus, lower cell density of the peripheral zone may contribute to active development of thyroid follicles. In regard to “niche theory”, central zone concentrated by mature thyroid follicles may be subjected to mature thyroid follicle-organized niche-like environment, whereas peripheral zone with sparse population of the mature follicles may lose the environment. In general, a niche environment for stem cell types maintains their resting state. Thus, the niche-like environment formed by the mature follicles may inhibit regeneration of the follicles at the center, while its loss at the peripheral zone may contribute to their regeneration.

We previously established thyroid tissue-organotypic culture system, as described above [6–8]. In our system, thyroid follicles actively regenerate at the peripheral part of the fragments, while the follicle regeneration never takes places at the central part. In fact, cellular growth at the peripheral part is extensively higher than that at the central part (Figure 6(a)). This phenomenon also takes place in an adipose tissue-organotypic culture system (Figure 6(b)) established by us [10–12]. On the basis of these facts, we propose the following possible theories for thyroid tissue regeneration: (1) cell density theory and (2) niche theory (Figure 6(c)). Firstly, we explain a “cell density theory”. Namely, the cell density of the central zone of the tissue fragments is prominently higher than that of the peripheral zone. In general, increased cell density in a microenvironment inhibits the regeneration and growth of cells that are subjected to contact inhibition of cell growth [13]. Thus, it is conceivable that decreased cell density of the peripheral zone may contribute to active development of thyroid follicles, supporting our previous study [10–12] that adipose tissue regeneration with active proliferation of preadipocytes and mesenchymal stem cells takes place at the peripheral zone, while this phenomena never occur at the central zone, using 3-D collagen gel culture of adipose tissue fragments. Secondly, we explain a “niche theory.” That is, mature thyroid follicles, which are concentrated in the central zone of thyroid tissue fragments, may organize a niche-like microenvironment for the follicles, while the microenvironment may be lost at the peripheral zone of the tissue fragments due to the loss of mature follicles. In general, a niche microenvironment for stem cell types maintains their resting state [14]. Thus, it seems likely that the niche-like microenvironment organized by mature follicles may inhibit the regeneration of follicles at the center, while its loss at the peripheral zone may contribute to their reproduction. In addition, the combination of “cell density theory” and “niche theory” may be involved in the active regeneration of thyroid follicles at peripheral zone of thyroid tissue fragments. Since these theories seem promising for explaining the mechanisms of tissue regeneration and remodeling, the regeneration studies regarding thyroid and adipose tissues are in progress in our laboratory.

To maintain body homeostasis, stem cells are considered to produce tissue-specific differentiating cell types (hematopoietic, intestinal, epidermal cells etc.) in response to daily cellular loss [14]. Partial defect of tissue is well known to initiate tissue regeneration such as liver regeneration after its partial defect by injury [15]. Thus, the defect of cell population and tissue is considered to be essential for the initiation of these phenomena. As described above, a tissue fragment is largely subdivided into the following two parts: (1) peripheral zone with lower density of cell population and (2) central zone with higher density of cell population (Figure 6(c)). On the basis of this fact, organotypic culture of tissue fragments seems to be a promising model for investigating tissue regeneration and remodeling *in vitro*. However, only several organotypic cultures of tissue fragments, including thyroid [6–8], brain [16, 17], adipose [10–12], and intestinal tissues [18] are successfully established. Thus, various tissues other than these tissues above should be applied to organotypic culture system. In addition, the injection of various stem cell types, including embryonic stem cells [19] and iPS cells [20, 21], into tissue fragments may allow us to study *in vitro* organogenesis with their proliferation and differentiation in a tissue microenvironment-dependent way. Since these issues are critical for regenerative medicine, further extensive studies are inevitably needed.

### 5.2. Models for Thyrocyte-Other Cell Type Interaction

A better understanding of the interactions between thyrocytes and other cell types such as C cells, fibroblasts, endothelial cells, and inflammatory cells seems critical for addressing the mechanisms of thyroid homeostasis and disorders, including autoimmune diseases and cancer. We here introduce some experimental models for challenging these issues.

#### 5.2.1. Thyrocyte-C Cell Interaction

For analyzing thyrocyte-C cell interaction, the above-mentioned thyroid tissue-organotypic system (Figure 4) is useful, because these cell types are localized within thyroid follicles* in vivo* and are difficult to culture C cells primary-isolated from the thyroid. Furthermore, the organotypic system can retain the viable two cell types within the follicles for a long term (Figures 4 and 5). In this system, we have neither detected the proliferative ability of C cells even in the stimulation of free calcium nor have addressed the interactions in detail [6–8]. This system will probably disclose many critical unresolved issues regarding the growth and differentiation of C cells, and thyrocyte-C cell interaction.

#### 5.2.2. Thyrocyte-Other Cell Type Interaction

**Figure 7:**
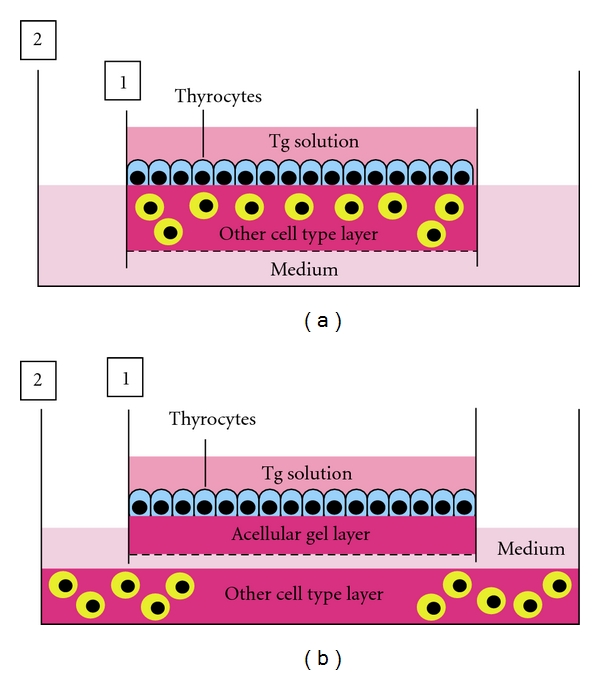
Models for analyzing thyrocyte-other cell type interaction. (a) Tg solution is overlaid on the surface of thyrocytes monolayer-cultured on other cell type-embedded collagen gel layer in an inner dish (1) with nitrocellulose membrane in its bottom. The inner dish is placed in a larger outer dish (2), and then culture medium is added to the outer dish. (b) To easily analyze the protein, mRNA, and DNA expression of both thyrocytes and other cell types under their interactions, the inner dish, in which Tg solution-exposing thyrocytes are cultured on acellular collagen gel layer, is placed in the outer dish where other cell types are cultured in a monolayer or 3-D collagen gel.

In general, thyrocytes contact with thyroglobulin (Tg)-containing colloid and ECM at the apical surface and the basal side, respectively. In addition, thyrocytes within thyroid follicles initially interact with the various stromal cell types at the basal side. For analyzing the interactions between thyrocyte and other cell types such as fibroblasts, endothelial cells, and inflammatory cells, the following model (Figure 7(a)) is useful. This model is organized as follows. First, each of the stromal cell types is embedded in collagen gel in an inner dish with a permeable nitrocellulose membrane in its bottom. After thyrocytes are seeded and monolayer-cultured on the gel layer, Tg solution is overlaid on the surface of thyrocytes cultured on the other cell type-embedded gel layer. The inner dish is placed in a larger outer dish, and medium is then added to the outer dish. This culture assembly simulates the *in vivo* integrated thyrocytes. To easily analyze the protein, mRNA and DNA expression of both thyrocytes, and other cell types under their interactions, the inner dish, in which Tg solution-exposing thyrocytes are cultured on acellular collagen gel layer, is placed in the outer dish where other cell types are cultured in a monolayer or 3-D collagen gel (Figure 7(b)). Given that the inner dish has a permeable nitrocellulose membrane in its bottom, various molecules can freely cross between the inner and outer dishes. For estimating the mechanisms of autoimmune diseases of the thyroid, analyses of the interactions between thyrocytes and macrophages or T and B lymphocytes will probably disclose the basic mechanisms of the initial immune crosstalk among these cell types.

Instead of normal thyrocytes, the utility of neoplastic thyrocytes in these models with or without Tg solution will allow us to investigate the mechanisms of the survival, growth, invasion, and metastasis of the cancer cells and cancer stem cells. In fact, we have demonstrated the apoptosis, growth, differentiation and invasion of various cancer cell types under the concept of cancer-stromal interaction, using these models [22–24]. Finally, this model is also useful for studies regarding the radiation biology of the thyroid, including radiation bystander effects [23].

Given that Tg concentration within thyroid follicles varies from 0.1 mg/mL up to 250 mg/mL [25, 26], this range of Tg concentration may be used in the experiment. In addition, 10 × 10^5^ of thyrocytes or the other cell types are suitable in these systems above (∗∗). The culture duration from 1 to 2 weeks would be enough to investigate the cell-cell interaction (∗∗).

### 5.3. Thyroid Tissue-Other Cell Type Interaction

**Figure 8:**
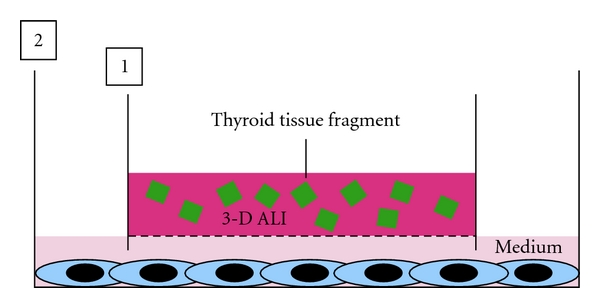
A model for analyzing thyroid tissue-other cell type interaction. Thyroid tissue fragments are embedded in ALI-treated collagen gel in an inner dish (1) with a nitrocellulose membrane. The inner dish is placed in a larger outer dish (1) in which each of other cell types is already cultured in monolayer or 3-D collagen gel. Then, culture medium is added to the outer dish.

For estimating the roles of thyroid tissue in biological behaviors of various cell types, for example, lymphocytes, cardiomyocytes, hepatocytes, adipocytes, osteoblasts, and nerve cells, thyroid tissue-based culture model is useful, because thyroid tissue-organotypic culture system can retain functional thyroid follicles with both thyrocytes and C cells for a long term. This model (Figure 8) is organized similarly to the model for studying thyrocyte-other cell type interaction. Thyroid tissue fragments are embedded in ALI-treated collagen gel in an inner dish with a nitrocellulose membrane. The inner dish is placed in a larger outer dish in which each of other cell types is already cultured. Using the similar model without ALI, we have demonstrated the active interactions between adipose tissue and renal tubular cells [27], osteoblasts [12], or cardiomyocytes [28]. In adipose tissue-tubular cell interaction, adipose tissue promotes the hypertrophy, polarization, and differentiation of tubular cells by attenuating their growth and apoptosis through opposing endocrine or paracrine effects of leptin and adiponectin, while tubular cells inhibit the regeneration of preadipocytes and mesenchymal stem cells [27]. In adipose tissue-osteoblast interaction, adipose tissue inhibits the proliferation and differentiation of osteoblasts, while osteoblasts prohibit the regeneration of preadipocytes and mesenchymal stem cells [12]. In adipose tissue-cardiomyocyte interaction [28], adipose tissue induces the lipotoxicity in cardiomyocytes, promoting the lipid accumulation and apoptosis of the cells together with the inhibition of their growth and differentiation. In turn, cardiomyocytes inhibit the regeneration of both MSC-like cells and preadipocytes from ATFs. Cardiomyocytes also promote the production of adiponectin from ATFs, while they inhibit that of leptin. Finally, an interesting study regarding adipose tissue-thyrocyte interaction is now going in our laboratory.

## 6. A New ALI-Based Classification of Culture System

ALI is a microenvironment of the skin, cornea, and respiratory and digestive tracts that are in continuity with the external environment atmosphere. The surface-lining cell types are situated at two-dimensional (2-D) ALI microenvironment. In solid organs, for example, thyroid, adipose tissue, liver, and kidney, various cell types are localized in the extravascular space that consists of ECM and tissue fluid percolated from blood vessels. The tissue fluid blended by nutrients and air molecules infiltrates into ECM and results in the formation of the moist stroma. This moist microenvironment seems to create that of ALI surrounding individual cell membrane. Thus, various cell types within solid organs would be situated at 3-D ALI microenvironment. In contrast, the microenvironment of intravascular space, which has the sufficient fluid of blood, is different from that of ALI-situated organs. Endothelial cells are thus situated at liquid-rich microenvironment, but not that of ALI.

**Figure 9:**
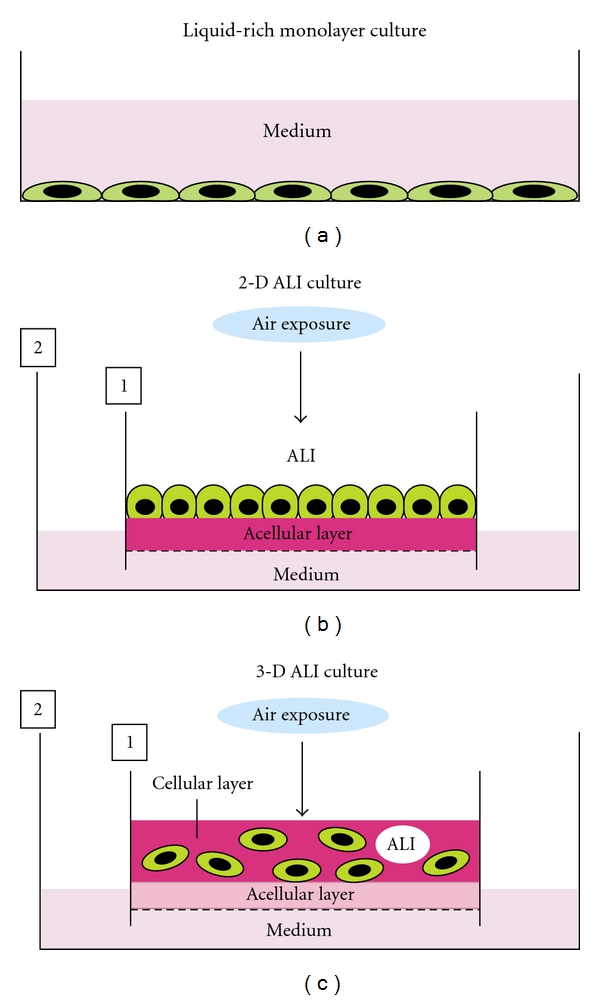
Scheme of a new classification of culture system. (a) Liquid-rich monolayer culture system. The usual monolayer culture under a submerged condition with enough medium is suitable for culturing the surface-lining cell types of endothelial cells, ependymocytes, renal tubular cells, and so on. (b) 2-D ALI culture system is useful for culturing the surface-lining cell types of epidermis, cornea, and respiratory and digestive tracts. (c) 3-D ALI culture is suitable for culturing parenchymal and stromal cell types of solid organs.

On the basis of our new concept of ALI, the microenvironments of many cell types of the body are subdivided mainly into the following three types. The first is the liquid-rich microenvironment of surface-lining cell types on which enough fluid is overlayed, for example, those of the cardiovascular system and cerebral ventricle. The second is the 2-D ALI microenvironment of surface-lining cell types on which sufficient liquid is not overlayed, for example, those of the skin, cornea, and respiratory and digestive tracts. The third is the 3-D microenvironment of parenchymal and stromal cell types of solid organs, for example, thyroid, adrenal, adipose tissue, liver, and kidney. We propose a new classification of culture systems (Figure 9).

## 7. Conclusions

For studying thyroid biology and diseases, we characterized monolayer, floating, 3-D collagen gel, and organotypic culture systems. Since these methods have their particular advantages and drawbacks, one can use each system in its specific way. We hope that these culture methods facilitate the studies of thyroid biology and disorders. Especially, experimental culture models described here will be probably a promising tool for disclosing many unresolved issues regarding normal and pathologic thyroid.
